# Perspective on plagiarism

**DOI:** 10.3402/jchimp.v2i1.18048

**Published:** 2012-04-30

**Authors:** Robert P. Ferguson, Marita Mike, Stephanie M. Griffin, Carole Lever

Plagiarism is the ‘action or practice of taking someone else's work, ideas, etc and passing it off as one's own’ (1). The word plagiarism was first recorded in the seventeenth century, and it originated from the Latin word plagiarius, which means ‘kidnapping’ (2). How close does a written piece have to be to another to be considered plagiarism? A match of more than 10% to other's existing works is one (3). This can occur in certain situations, for example, according to Segal et al. and others, more than 5% of those applying to a prestigious Northeastern United States teaching hospital exceeded 10% similarities when writing personal statements. This appears particularly problematic for international medical graduates. In a recent survey of internal medicine community hospital programs that we conducted through the Community Hospital Education and Research Network (CHERN), more than one-half of the replying programs were dominated by English as a Second Language (ESL) residents – 82%.

Plagiarism is related to intellectual property, which is legally defined as ‘creation of the mind-creative works or ideas embodied in a form that can be shared or can enable others to recreate, emulate, or manufacture them. There are four ways to protect intellectual property- patents, trademarks, copyrights, or trade secrets’ (4). ‘Copyright is a form of protection provided by the laws of the United States (title 17, U.S. Code) to the authors of “original works of authorship”, including literary, dramatic, musical, artistic and certain other intellectual works’ (5). Plagiarism in and of itself is not a legal ‘term of art’ with regard to intellectual property protection. It is defined in dictionaries, and as above, more than one definition may apply. Punishment/restitution with regard to plagiarism is not legally codified as an intellectual property right, although in some situations copyright infringement may have simultaneously occurred and restitution for copyright infringement may be sought.

In the most basic terms – copyright protection protects against ‘copying’ a protected work. Copyright does not protect against using someone else's idea to produce the same ‘work’ (6). For example, a famous photographer may take a photograph, which becomes widely published. It would be a copyright infringement to photocopy and distribute that photograph. It would not be a copyright infringement to go to the same spot, set your camera to the same settings, attempt to take the same photograph, make copies, and sell them. The image is the protected work and it was not copied – the image in this example was independently reproduced.

Those who do not speak English first may struggle with grammar and syntax. Writing may be more difficult for them, both reporting scientific results and writing personal statements. Poorly written manuscripts lend themselves to a negative interpretation of the content of what is written. In addition, these individuals may not have a clear understanding of what plagiarism is and what rules of intellectual property are in the United States, especially if coming from a non-western culture. Some cultures consider copying another's words a form of flattery (7). In many situations, the ESL plagiarizer may not appreciate the rules of American academia (8).

*Journal of Community Hospital Internal Medicine Perspectives* (JCHIMP) staff encountered plagiarism in 2011. It occurred the first time we had called for papers for the JCHIMP. A reviewer had noted a sentence that was out of style with the rest of the paper, that is, it was much better. The reviewer dutifully ‘Googled’ the entire sentence and found it exactly the same as in a report published 8 years earlier in another English-speaking country on the same topic. The student happened to be an observer in our department when this was picked up. He was called in and asked about the authorship of the sentence in question. He admitted that he had copied the sentence from the other journal. The manuscript was rejected, and we canceled his observer status.

There is a fine line in calling something plagiarism. For example, I recently noted a phrase published in an editorial about the 2010 outbreak of *Escherichia coli* in Europe related to sprouts, and the phrase by Blaser was ‘organisms armed for mayhem’ (9). A similar toxic organism, *Neisseria meningitidis*, was described in a recent publication of JCHIMP of two fatal cases of meningococcemia, including one with autopsy-proven Waterhouse-Friderichsen syndrome (10). ‘Armed for mayhem’ came to mind but ultimately the authors decided not to use it because it was comparing apples to oranges; *E. coli* to *N. meningitidis*. They changed ‘armed for mayhem’ to ‘rendering havoc in individual cases’. They decided to Google ‘*E. coli* armed for mayhem’ anyway and came up with numerous quotes in the lay and medical literature after the outbreak was reported in the *New England Journal* issue cited above. This quote would have been, if not cited, a case of plagiarism, even if it was only a few words. According to the Committee on Publication Ethics (COPE) (11), a few words can be plagiarizing, although this is not as serious an offence when compared to lifting an entire sentence or paragraph. This is a difficult area.

Dealing with potential plagiarism problems requires enforcement and education, using the available technology. There are many tools available that help detect, prevent, and teach plagiarism. The Purdue Online Writing Lab (OWL) (www.owl.english.purdue.edu/owl) is widely used by researchers and those teaching research, as it includes the basics of writing, plagiarism, style guides, and much more. Plagiarismchecker.com and paperrater.com, two free resources, perform basic plagiarism assessment, while eTBLAST (www.etblast.org) searches medical literature databases such as PubMed, Clinical Trials. The Office of Research Integrity (http://ori.hhs.gov) website is a wealth of information for plagiarism, self-plagiarism, copyright, and citing structure. Many researchers and educators point to the need to educate less on technology and more on the finer points of the research process. Tools are available to detect originality in articles, but there are no tools for determining one's ethics. As part of the ongoing teaching curriculum, it is our responsibility to instill publication, copyright and plagiarism education, and ethics education. Software plagiarism programs include TurnitinAdmissions.com, iThenticate.com, and Copyscape.com. Here at JCHIMP we use iThenticate.com. This allows us to take a submission and run it through the program to see if any part of the manuscript is plagiarized. We receive a report that allows us to see what percentage of the submission is not authentic. The user may receive a report that says that 30% of the document was plagiarized, but it is broken down into smaller sections, and the highest percentage may only be 5%. These percentages can also be a mistake on the writer's part; an example is the writer may just have forgotten to put quotations on a phrase. The disadvantage with these software programs is that even with the report, what percentage tells the user there is a problem? There is no such thing as a ‘magic number’ that will tell you whether a document contains problematic content (12). Another way to detect plagiarism is by taking a part of a document and running it through Google. Google will allow you to pull up any other documentation that is similar.

Peer review was initiated in scientific journals in the mid-1770s when the editors of the Philosophical Transactions of the Royal Society of London launched the peer review system to prevent authors from plagiarism. It was two centuries later when journal editors found it necessary to have peer reviewers evaluate manuscripts. Their two functions are to ensure that work is published only if it meets appropriate standards of scholarship and methodology, and it helps authors improve their manuscript ‘… assessing the manuscripts for originality, importance, validity and clarity …’ (13).

Educating residents as far as the definitions of plagiarism and intellectual property should be a priority. We are planning to do this year with our new residents during orientation, that is, encouraging scholarly work while educating residents on plagiarism. We will give them cultural orientation including the definitions of plagiarism and how plagiarism is now picked up through technology. We will demonstrate the technology.

We will continue to patrol all submissions to JCHIMP for plagiarism. We must be mindful that copying may be subconscious rather than an advertent act. We plan to teach plagiarism avoidance (3). In retrospect, we could have done better when confronting our plagiarizer. Perhaps, he did not fully understand the consequences of what he did.

Elsewhere in this issue there are 13 papers. There are three original research studies: first, the use of a new computerized model to improve patient management of Chronic Obstructive Pulmonary Disease (COPD) (14); second, a resident initiated plan to reduce re-admissions of congestive heart failure (15); and, third, a study showing the important differences in dosing for replacement of vitamin D (16). There are five case reports. There is a brief report on the use of absolute lymphocyte count on admission to rapidly predict pneumocystis pneumonia in patients not previously known to have HIV (17). There is also our imaging column, which is on aspergillus infections in the lung (18) and an Electrocardiogram (EKG) case with pulsus alternans (19). There is also the first in our *History of Medicine series* on the evolution of the 12-lead EKG (20). Also, there is a report on the important issues related to obstetrics and gynecology clinical faculty in community hospital programs (21). We feel that this topic is of interest to our readership because teaching faculty in community hospitals is a central issue of JCHIMP, regardless of the specialty.

*Robert P. Ferguson, MD* Chief of Medicine*Marita Mike, MD* Consulting Lawyer*Stephanie M. Griffin, BBA* Fellowship Coordinator*Carole Lever* Library Manager
